# MemBrain: An Easy-to-Use Online Webserver for Transmembrane Protein Structure Prediction

**DOI:** 10.1007/s40820-017-0156-2

**Published:** 2017-09-27

**Authors:** Xi Yin, Jing Yang, Feng Xiao, Yang Yang, Hong-Bin Shen

**Affiliations:** 1 Institute of Image Processing and Pattern Recognition, Shanghai Jiao Tong University, Shanghai, 200240 People’s Republic of China; 2 Key Laboratory of System Control and Information Processing, Ministry of Education of China, Shanghai, 200240 People’s Republic of China; 3 Department of Computer Science, Shanghai Jiao Tong University, Shanghai, 200240 People’s Republic of China; 4 Key Laboratory of Shanghai Education Commission for Intelligent Interaction and Cognitive Engineering, Shanghai, 200240 People’s Republic of China

**Keywords:** Transmembrane α-helices, Structure prediction, Machine learning, Contact map prediction, Relative accessible surface area

## Abstract

Membrane proteins are an important kind of proteins embedded in the membranes of cells and play crucial roles in living organisms, such as ion channels, transporters, receptors. Because it is difficult to determinate the membrane protein’s structure by wet-lab experiments, accurate and fast amino acid sequence-based computational methods are highly desired. In this paper, we report an online prediction tool called MemBrain, whose input is the amino acid sequence. MemBrain consists of specialized modules for predicting transmembrane helices, residue–residue contacts and relative accessible surface area of α-helical membrane proteins. MemBrain achieves a prediction accuracy of 97.9% of *A*_TMH_, 87.1% of *A*_P_, 3.2 ± 3.0 of *N*-score, 3.1 ± 2.8 of *C*-score. MemBrain-Contact obtains 62%/64.1% prediction accuracy on training and independent dataset on top *L*/5 contact prediction, respectively. And MemBrain-Rasa achieves Pearson correlation coefficient of 0.733 and its mean absolute error of 13.593. These prediction results provide valuable hints for revealing the structure and function of membrane proteins. MemBrain web server is free for academic use and available at www.csbio.sjtu.edu.cn/bioinf/MemBrain/.

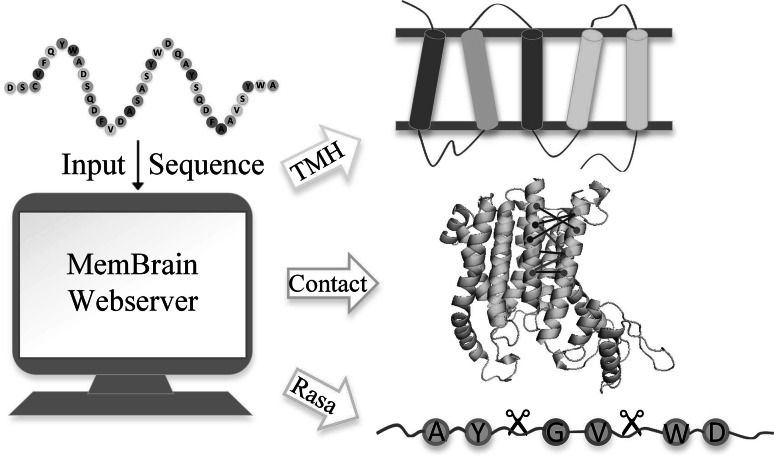

## Highlights

- MemBrain is a fully automatic online tool for transmembrane protein structure prediction, which is able to predict the irregular half-transmembrane helix.
- MemBrain’s theoretic predictions provide timely and important clues for further wet-lab experiments.

## Introduction

**Fig. 1:**
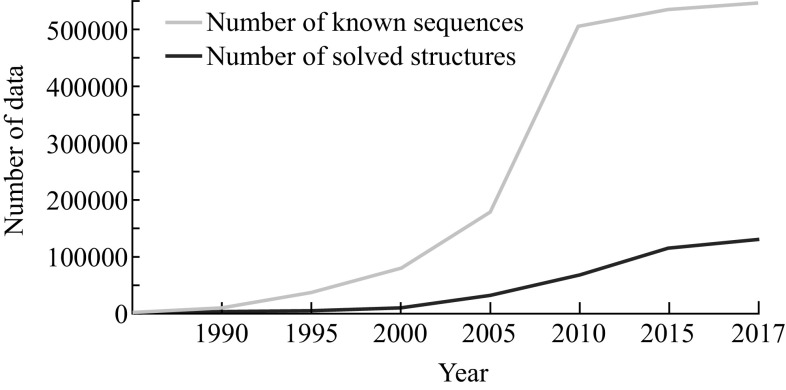
The gap between known protein sequences and structures is rapidly expanding

Significant advancement of sequencing technologies has resulted in an explosion of protein amino acid sequences in various databases such as the UniProt (as shown in Fig. 1). However, due to the difficulties of wet-lab experiments, the gap between the numbers of known sequences and their corresponding experimentally solved structures keeps growing [1]. Thus, the development of the fast and accurate computational approaches for predicting structures from the amino acid sequences has attracted more and more attention. Membrane proteins constitute approximately 30% of the proteins in both prokaryotic and eukaryotic genomes [2], due to the crucial functions of them, and more than 60% current drug targets are membrane proteins [3]. The 3D structures of membrane proteins will provide important insights for membrane protein-orientated drug design. For instance, the binding mechanisms of membrane protein-drug ligand can be modeled with the 3D structures. However, solving membrane protein structures through the wet-lab experiments is extremely difficult. The reason is that membrane proteins usually have one or more transmembrane segments, which are very hydrophobic making the chances for crystallization of membrane proteins small [4, 5]. In such a case, computational bioinformatics algorithms are highly desired, which will provide fast and accurate membrane protein structure predictions.

**Fig. 2:**
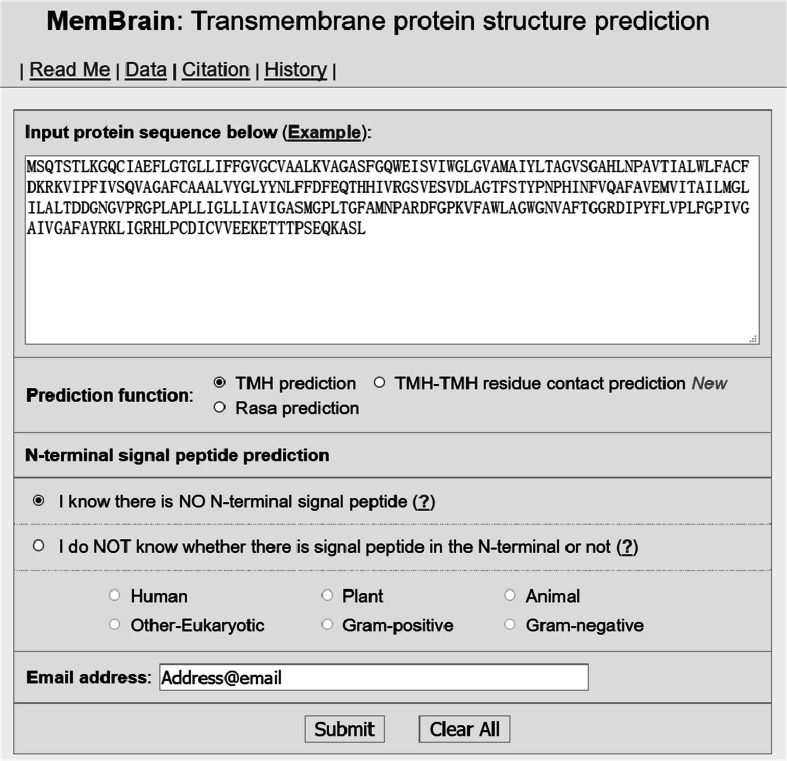
A screenshot of the submission interface of MemBrain web server (www.csbio.sjtu.edu.cn/bioinf/MemBrain/)

For the past 10 years, we are developing an online predictor named MemBrain (as shown in Fig. 2) that can predict α-helical membrane protein structure [6–8]. Currently, this predictor consists of the following three functional modules:

### MemBrain-TMH: Transmembrane α-Helical Segment (TMH) Prediction

A TMH is a segment of residues along the sequence which spans the membrane. The prediction of TMHs is labeling the residue positions of inside/outside membrane. A large portion of the membrane proteins are transmembrane proteins, which have one or multiple hydrophobic transmembrane segments. Transmembrane proteins have two types: α-helical and β-barrels proteins. The former proteins are the major membrane proteins and the latter one only account for ~30% in membrane proteins. We also developed a method for predicting spanning segments for β-barrels [9]. One of the important steps for the membrane protein structure prediction is to identify the transmembrane segments from the amino acid sequence, e.g., TMH. The initial methods of TMH structure prediction employed the amino acid hydrophobicity analysis; later, benefitting from the rapid expansion of structural database, machine learning methods have been widely applied to automatically learn the rules for classifying the TMH residues from the solved structures (training samples). Such TMH topology structure predictors include HMM-based approach like TMHMM [10], SVM-based methods like SVMtm [11], the OET-KNN-based MemBrain [6], etc. The prediction of irregular half TMHs is a challenging topic in the transmembrane TMH predictions. In our MemBrain-TMH model, the multi-scale modeling and dynamic threshold approach are incorporated to improve its prediction performance.

### MemBrain-Contact: Residue–Residue Contact Map Prediction

When two residues are close enough in the space (e.g., <8 Å), they are generally acknowledged as ‘contact.’ The contact map prediction is to generate a 2D map marking the contacted residue pairs. Although the TMH structure predictions can help figuring out the general structure topology of α-helical membrane protein, it is not enough to build the 3D structure of a membrane protein. The residues contact map provides spatial constraints for constructing tertiary structure models of TMH proteins, which has recently been a hot topic in protein structure prediction [12–15]. The existing methods for predicting residue–residue contacts of α-helix proteins and TMH–TMH interactions from the primary sequences can be generally divided into two categories: (1) machine learning-based methods, (2) statistical-based coevolution mining methods. Our results show that these two branches of methods highly complement each other [7]. The machine learning-based engines need the training process and highly depend on the distributions of training dataset. Hence, the prediction outputs of machine learning-based models have higher preference to match the distribution of the training set, resulting in a relatively lower generalization and coverage of the predictions. Training process is not needed in the coevolution mining methods, which align the query sequence against a large protein sequence pool to calculate the residue pair potential coevolution score. And because such statistical approaches are unsupervised methods, they will have predictions of wider coverage, but with higher false positives at the same time. Our MemBrain model is a consensus predictor of the two branches of engines, so its prediction accuracy is higher than a single independent model.

### MemBrain-Rasa: Residue Relative Solvent Accessibility Surface Area (Rasa) Prediction

In a 3D structure, some residues are buried into the internal core making them hard to be reached by other ligands. The relative solvent accessibility is a quantitative measurement of the visibility of the residues in a structure. Although many computational methods have been developed to predict the residues’ Rasa in soluble proteins [16, 17], relatively few approaches are available for the membrane proteins. The reason is that the solved membrane protein structures are much fewer than the soluble proteins, making the training samples difficult to collect. The module of MemBrain-Rasa software is a combination of machine learning-based engine and the segment template-based module, which can solve the prediction preference problem caused by the pure machine learning-based model.

## MemBrain Prediction Functions

### MemBrain-TMH: Prediction of TMHs in Membrane Proteins

Accurate TMH prediction is a long-term interest in transmembrane protein structure prediction. At the very beginning of methodology development in this problem, motivated by the fact that transmembrane residues are usually highly hydrophobic, average hydrophobic scores were used for detecting the hydrophobic segments. Later, more studies have revealed that this task is much more complicated than initially thought. For instance, very short (<10 residues) and very long (>35 residues) irregular TMH helices have been found and some loop regions linking the neighboring TMH segments can be very short (e.g., ~2 residues). These structure complexities have posed significant difficulties for prediction methodology development.

**Fig. 3:**
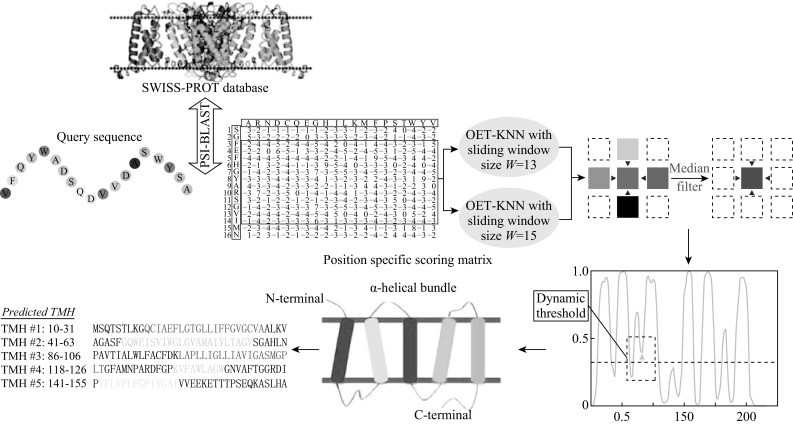
The pipeline of MemBrain for predicting transmembrane α-helices. For a query sequence, we generate the position specific scoring matrix as input features by searching against SWISS-PROT database using the PSI-BLAST tool. The OET-KNN algorithm is employed as the classifier with fused different sizes of sliding window for extracting features. Median filter is applied to smooth the profile of predicted probabilities. Finally, the dynamic threshold is effectively used to optimize the results of prediction

In our MemBrain-TMH module (as shown in Fig. 3) [8], two typical strategies are adopted to enhance the TMH predictions.

#### Multi-scale Predictors Modeling

The input features are amino acid evolution information from optimized sliding windows with different lengths. We built a profile for a query sequence with *L* residues by the position specific scoring matrix (PSSM) implemented by PSI-BLAST [18] program. The PSSM contains amino acid evolutionary information from multiple sequence alignment searching against the SWISS-PROT database [19]. The profile has *L* rows and 20 columns, where the *i*th row represents the probabilities of the *i*th residue in the protein sequence being mutated to 20 native residues during the evolution process. The sequence evolution knowledge encoded in the PSSM helps to remove the potential noise caused by mutations.

Considering the irregular lengths of the TMH, we designed the multi-scale model with different sliding window sizes. The size of the sliding window for extracting input feature has a great impact on the prediction outcome. If the sliding window is too small, the prediction accuracy would suffer from the loss of neighborhood sequence information; on the contrary, if it is too large, much redundant information will be included especially for the cases of short TMHs. We tried different lengths of windows for fusing the global and local sequence parameters, and at last we combined two window sizes to minimize the bias induced by a single window size, i.e., *W* = 13 and *W* = 15. This strategy makes current MemBrain approach capable of predicting half TMHs or tight turns shorter than 15 residues. The MemBrain also employs a powerful machine learning technique, the optimized evidence-theoretic K-nearest neighbor (OET-KNN) algorithm, which will output a propensity of residue belonging to TMH segments. The final obtained TMH propensity is averaged over the results of lengths 13 and 15 for each residue along the sequence.

#### Dynamic Threshold Decision

For a query sequence, a plot of predicted TMH propensity scores gives an overview of the residue-specific TMH propensity. In order to optimize the accuracy, we adopt the median filter technique to smooth the predicted TMH propensity profile for reducing noise and avoid the burr phenomena. The final TMHs are determined by the smoothed propensity plot. A threshold will be needed for classifying them into TMHs or non-TMHs, i.e., if the predicted scores of residues are higher than the threshold, they are predicted as TMH residues. A fixed threshold is often used for this purpose, which may be problematic for segmenting two TMHs linked by short loops.

Many high-resolution membrane protein 3D structures have shown that two adjacent TMHs could often be connected by very short loops, e.g., <2 residues. In such cases, the predicted TMH propensity scores corresponding to the short loop residues will also be very high due to the sliding window technique used for extracting features. Taking *W* = 13 as an example, if the short loop is composed by 2 residues, then 11 residues belong to TMH in the window making the TMH features dominate for loop residues. Therefore, the contiguous TMH segments linking with short loops or tight turns are often misclassified as a long one. This indicates that the optimal threshold for defining two TMHs separated by long loops is very different from the threshold required for identifying TMHs separated by short loops. To solve this problem, we exploit the dynamic threshold strategy for identification of TMHs from the propensity scores. First, we set an initial threshold as 0.4, i.e., residues with propensity greater than or equal to 0.4 are considered as TMH. Second, we gradually increase the initial value of *T* with step size of 0.05 up to find the plot valley to decide whether we need to split the initial segments into two by a set of pre-learned rules. The results show that the dynamic threshold method not only improves the localization prediction of THM residues, but also enhances the correct number of TMH predictions.

### MemBrain-Contact: TMH–TMH Residue Contact Map Prediction

**Fig. 4:**
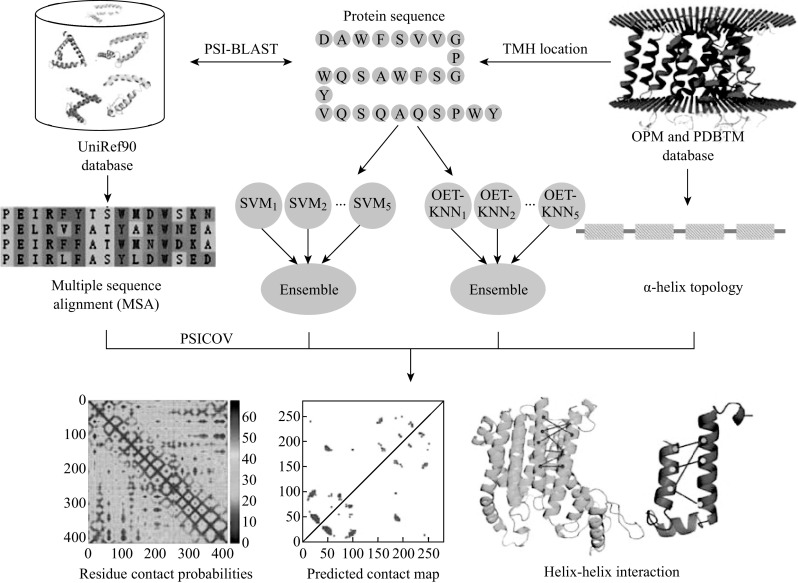
The pipeline of MemBrain-Contact for predicting TMH–TMH contact map. We extract the TMH locations and topologies from protein database to build the training dataset. The coevolved mutation analysis by PSICOV using multiple sequence alignment generated by PSI-BLAST and machine learning-based algorithm outputs are combined to generate the final predictions

Based on the determined TMHs, the prediction of TMH–TMH residue contacts can provide crucial spatial constraints for accurately modeling tertiary structures of membrane proteins [15, 20]. The MemBrain-Contact prediction module is constructed by combining statistical machine learning algorithms and biological residue coevolution analysis from multiple sequence alignments as shown in Fig. 4 [7]. The machine learning-based prediction algorithm was implemented by applying multiple random under-samplings so that strong diversities can be produced via different learning methods in various spaces. The coevolved mutation scores from multiple sequence alignments were generated by PSICOV algorithm [12].

Fusing the coevolution-based engine and machine learning-based engine is a typical advantage of MemBrain-Contact module. We found that these two engines highly complement to each other. The coevolution-based engine does not need the training process, which is an unsupervised approach and hence can result in a wide coverage of predictions but with relatively high false positives. The machine learning-based engine is a supervised learning approach, whose outputs are dependent on the training samples, and hence has a relatively low coverage of predictions. The combination of the two approaches will not only improve the prediction coverage but also reduce the false positives, resulting in an overall performance improvement.

### MemBrain-Rasa: Relative Accessible Surface Area Prediction

**Fig. 5:**
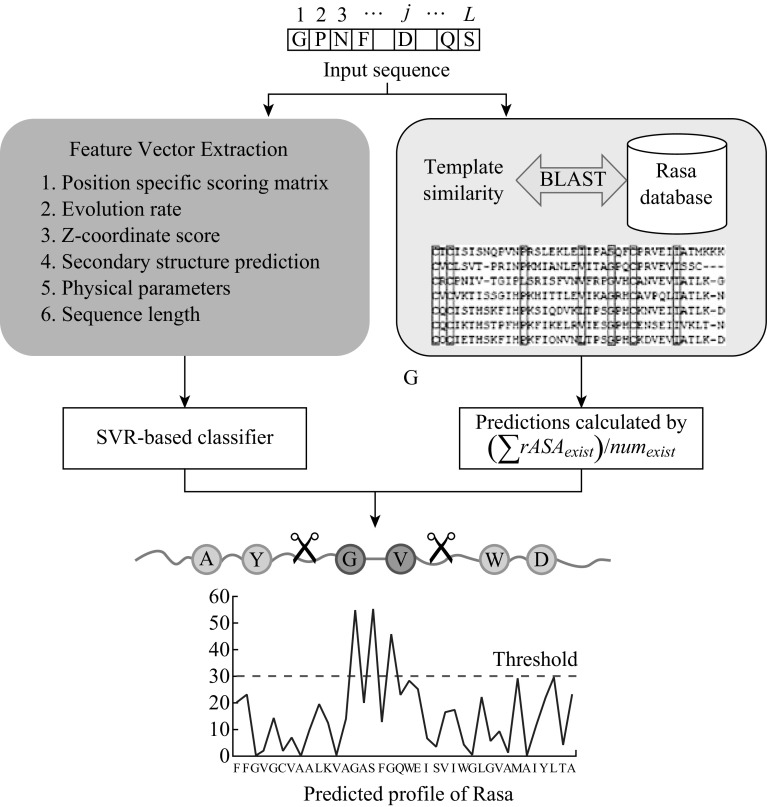
The flowchart of MemBrain-Rasa prediction protocol. For a protein sequence, we extract six kinds of sequential features, which will be fed into the SVR classifier. We also designed a segment template similarity-based prediction engine for searching similar segments as templates for the target sequence against a locally constructed structure data pool. The outputs of the two engines are combined together to improve the prediction of relative accessible surface area

Prediction of RASA in α-helical transmembrane proteins provides the relative accessibilities of the residues which are helpful to 3D structure prediction. In MemBrain-Rasa, we designed a segment template similarity-based prediction engine, which is effectively combined with the machine learning engine to improve the performance. In order to take the advantage of the solved structures, we organized a local database of residue relative solvent accessibility surface area from the protein data bank (PDB), which is applied to search similar segments as templates for the target sequence against the local data pool. The template similarity-based prediction is then fused with the output of support vector regression (SVR) using a designed knowledge rule. Figure 5 shows the MemBrain-Rasa prediction protocol [8].

A typical merit of MemBrain-Rasa is its hierarchical prediction model by combining supervised SVR model with a segment template similarity-based approach as the whole computational framework to deal with RASA prediction problem. The results show that for many long protein sequences, it is very hard to find homology structure templates of the full chains. However, when we only consider short segments, many existing structure templates can be found, which provide important complement to the pure machine learning-based predictions.

### Prediction Performance of MemBrain

On a test dataset including 70 helical membrane proteins consisting of 378 TMHs, MemBrain achieves a prediction accuracy of 97.9% of *A*_TMH_, 87.1% of *A*_P_, 3.2 ± 3.0 of *N*-score, 3.1 ± 2.8 of *C*-score, where *A*_TMH_ denotes the rate of correctly predicted TMHs, *A*_P_ denotes the ratio of correctly predicted proteins (all predicted TMHs are successful), and *N*-score and *C*-score are the accuracy scores of predicted ends of TMH segments.

Two benchmark datasets are used to evaluate the performance of MemBrain-Contact module, i.e., a training dataset consists of 60 α-helical proteins and an independent dataset with 21 α-helical proteins. Both of the two datasets have a sequence identity cutoff at 40% among pairwise sequence for reducing protein homology similarity redundancy. Their TMH locations and native topologies were extracted from the databases of TOPDB [21], PDBTM [22] and OPM [23]. For top *L*/5 contact predictions, prediction accuracies are 62%/64.1% on the training and independent datasets, respectively, where *L* is the length of sequence. The experimental results on 13 solved G protein-coupled receptors have shown that the predictions of MemBrain-Contact engine have helped increase the TM-score of the I-TASSER models by 37% in the transmembrane region.

On a benchmark dataset consisting of 52 membrane proteins composed of 80 chains with pairwise sequence identity <20% to avoid homology redundancy, the MemBrain-Rasa achieves a Pearson correlation coefficient of 0.733 and mean absolute error of 13.593, which are significantly enhanced compared to either the machine learning-based or template-based engines.

## Conclusions and Future Development

MemBrain is a fully automated online server and is free to academic use, which is available at http://www.csbio.sjtu.edu.cn/bioinf/MemBrain/. For a query protein, the user simply needs to input its amino acid sequence and select the corresponding prediction functions, and then submit it to the server. Prediction results will be sent back to the user’s email address when the task is finished. Usually, MemBrain is very fast, depending on the length of protein sequence, and it will automatically send back the results in 5 min of most cases. MemBrain theoretic predictions have provided useful information to the wet-lab studies of membrane proteins [24–26].

In the future, we will keep on updating MemBrain to make it more powerful. One of the potential directions is developing the deep learning-based modules, which are expected to be highly complementary to current engines. Deep learning algorithms represent a new progress in the statistical machine learning field [27, 28] which is expected to provide more opportunities for further enhancing the prediction performance of MemBrain.
